# Aircraft Image Recognition Network Based on Hybrid Attention Mechanism

**DOI:** 10.1155/2022/4189500

**Published:** 2022-04-18

**Authors:** Yanfeng Wang, Yinan Chen, Runmin Liu

**Affiliations:** ^1^College of Systems Engineering, National University of Defense Technology, Changsha 410082, China; ^2^College of Computer & Information Engineering, Central South University of Forestry and Technology, Changsha 410004, China; ^3^College of Sports Engineering & Information Technology, Wuhan Sports University, Wuhan 430079, China; ^4^AiTech Artificial Intelligence Research Institute, Changsha 410000, China

## Abstract

With the deepening of deep learning research, progress has been made in artificial intelligence. In the process of aircraft classification, the precision rate of aircraft picture recognition based on traditional methods is low due to various types of aircraft, large similarities between different models, and serious texture interference. In this article, the hybrid attention network model (BA-CNN) to implement an aircraft recognition algorithm is proposed to solve the above problems. Using two-channel ResNet-34 as a characteristic extraction function, the depth of network is increased to improve fine-grained characteristic extraction capability without increasing the output characteristic dimension. In the network to introduce a hybrid attention mechanism, respectively, between the residual units of two ResNet-34 channels, channel attention and spatial attention modules are added, more abundant mixed characteristics of attention are obtained, space and characteristics of the local characteristics of the channel response are focused, the characteristics of redundancy are reduced, and the fine-grained characteristics of learning ability are further enhanced. Trained and tested on FGVC-aircraft, a public fine-grained pictures dataset, the recognition precision rate of the BA-CNN networks model reached 89.2%. It can be seen from the experimental results, the recognition precision rate of the original model is improved effectively by using this method, and the recognition precision rate is higher than most of the existing mainstream aircraft recognition ways.

## 1. Introduction

Aircraft picture recognition has been a research hotspot of fine-grained picture recognition of machine vision field. In recent years, with the continuous deepening of artificial intelligence, aircraft recognition ways based on deep learning have been adopted in airport management, military, and other fields. Aircraft picture recognition is a typical representative of fine-grained picture classification. The fine-grained nature of aircraft pictures leads to large interclass [1] similarity and intraclass variability among aircraft models, which in turn affects the precision rate of aircraft classification. How to effectively use the information about aircraft pictures and research a better performance aircraft recognition algorithm is not only a certain promotion significance of the application of aircraft recognition system but also a certain reference values for the solution to other picture recognition problems [2].

The current fine-grained aircraft picture recognition algorithms mainly include two directions: one is the recognition algorithm based on traditional picture processing and the other is the recognition algorithm based on deep learning. The recognition algorithms based on traditional picture processing mainly include template matching algorithm [3] and traditional characteristic extraction recognition algorithm [4]. The template matching algorithms mainly analyzes the regions of the target picture, compares the characteristics of each region with those of the template, and determines the category of the target picture according to the degree of similarity. The template matching algorithm requires low contrast of the picture and has a good ability to deal with the presence of occlusion in the picture, but it has the disadvantages of large computation and poor real-time performance. Since traditional picture recognition algorithms often have difficulty in finding high-quality characteristics, resulting in low recognition precision rate, the mainstream research direction is currently based on deep learning recognition methods, which mainly use CNNs that are well suited for processing two-dimensional picture data, such as Chevalier et al. [5] proposed a deep learning-based LR-CNN network model for picture classification, Huihui Li [6] proposed a PCNN network model for aircraft classification, Malekzadeh [5] proposed a DNN network model for extracting aircraft characteristics, Ting-Bing Xu [7] proposed an “end-to-end” FCN for fast aircraft classification, Tsung-Yu Lin et al. [8] proposed a B-CNN network model.

Although the deep learning way has been utilized to obtain better aircraft recognition results than traditional methods, the current deep learning method has a single network structure, which ignores the channel and spatial relationships that aircraft pictures have, resulting in a certain loss of information and hindering further improvement in recognition precision rate. Moreover, the current convolutional neural network structure dealing with recognition problems of aircraft pictures is to train separate networks according to each input, and then through each separate network, the recognition prediction is performed for each input separately. After analysis, it is known that this method has two main problems: on the one hand, the number of networks used is too many and unrelated to each other, thus increasing the cost of network training and causing information redundancy, resulting in time inefficiency; on the other hand, the inherent relationship between aircraft picture channels and space is ignored, which limits the improvement in recognition results.

Thereby, this article proposes a hybrid attention network model (BA-CNN) to implement the aircraft recognition algorithm.

The dedications to this article are as follows:

1. Using two-way ResNet-34 as the characteristic extraction function, adding the depth of the network to improve the fine-grained characteristics extraction capability without adding the output characteristic dimension
2. A hybrid attention mechanism is introduced into the network to add the channel attention module and spatial attention module between the residual units of two ResNet-34 channels respectively to obtain richer hybrid attention characteristics, focus on local characteristic channels and spatial response parts in the characteristic map, reduce characteristic redundancy, and further enhance the fine-grained characteristics learning capability at the same time

The recognition precision rate of the BA-CNN network models reaches 89.2% when trained and tested for the publicly available fine-grained picture dataset FGVC-aircraft. Experimental consequences showed that the recognition precision rate of the original model is improved effectively by using this method, and the recognition precision rate is better than most of the existing mainstream aircraft recognition ways.

## 2. Materials and Methods

### 2.1. Data Acquisition

**Figure 1:**
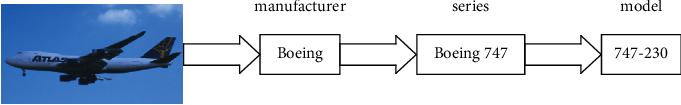
Sample labeling diagram.

Most of the picture recognition network models are required to have a certain size of dataset to optimize the algorithm. For the aircraft recognition problem, the pictures and labels used in this article are mainly collected from the FGVC-aircraft [9] dataset, which contains 10,000 aircraft pictures, with the size of every picture ranging from 33 KB to 1 MB. Each aircraft picture is uniquely labeled with “manufacturer,” “series,” and “model.” As shown in Figure 1, this aircraft picture is labeled as the 310-300 model of the Airbus 310 series designed by the airbus manufacturer.

**Figure 2:**
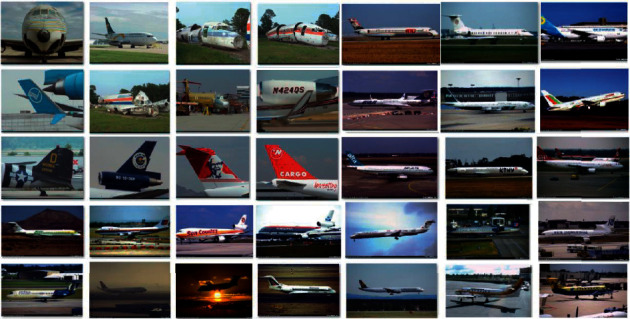
Selected aircraft sample pictures.

Figure 2 shows some of the aircraft pictures in the dataset used in this article. For the aircraft dataset in this article, when aircraft is considered as a large category, they are an object of high intra-class similarity, and the objects of its class all have the basic structure of an aircraft (e.g., fuselage, wings, and engines), so it is inherently difficult to subdivide them internally. It is easier to classify various aircraft from different manufacturers compared to classifying various types of aircraft produced by the same manufacturer. This is because aircrafts produced by the same manufacturer are similar in structure and appearance, thus making recognition more difficult. In addition, factors such as complex backgrounds and aircraft crippling can also affect the precision rate of the classification. In order to obtain the reliable experimental consequence and improve the applicability to the multi-label recognition problem, manufacturers with multiple types of aircraft were selected in the construction of the dataset, while the number of different label data was appropriately balanced, and 7000 pictures were randomly selected from them to form the train set; however, the remaining 3000 pictures were used as the test set, so that the experiments can accurately test the effectiveness of the algorithms in this article.

Considering the quality and quantity of the pictures in the dataset, the dataset is expanded and some of the pictures are enhanced, and the aircraft pictures are preprocessed in a specific way to heighten the recognition of the pictures and protrude the aircraft part of the pictures. In order to facilitate the learning optimization of the algorithmic network in this article, taking into account the needs of the algorithms used in this article. The dataset plays a top-down role in the solution to the whole recognition problem, and it is directly related to the specific representation of the aircraft recognition problem and the structure of the algorithm for solving the problem.

**Figure 3:**
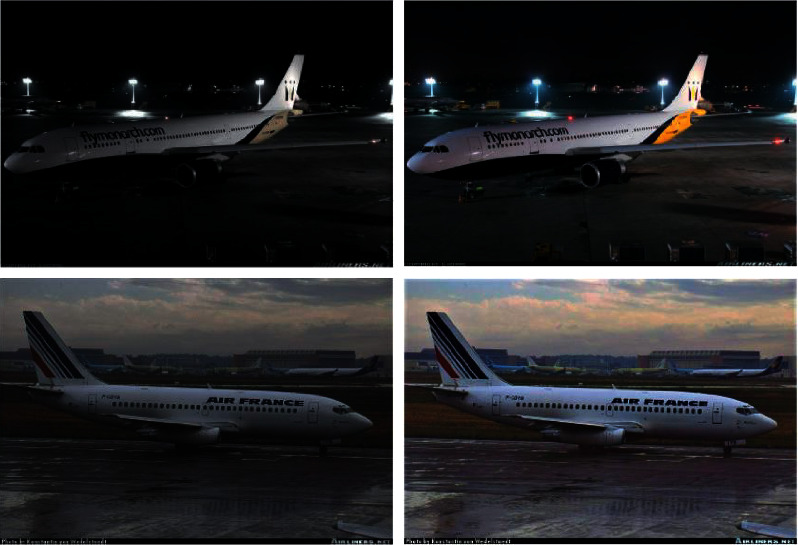
Aircraft pictures before and after spatial domain enhancement.

In this article, we mainly use the spatial domain enhancement method to sharpen the relatively blurred and shadowy aircraft pictures and increase the contrast of the pictures. The spatial domain method is mainly a direct operational processing of pixel grayscale values in the spatial domain, and the more common methods include gray-level transformation, histogram correction, picture space smoothing, and sharpening processing. Grayscale transformation mainly uses the mapping function to change the gray-level range of the picture, which can be corrected for a certain part of the picture or the whole picture underexposed, in order to strengthen the gray contrast of the picture; histogram correction by using a specific conversion function to change the gray distribution of the picture, so that the high and low brightness areas of the picture gray map have approximately the same intensity, which can make the picture with the desired gray distribution, so as to selectively highlight the desired gray distribution. Thus, the desired picture characteristics are selectively highlighted to meet the needs of a specific task.  Figure 3 shows an aircraft picture before and after spatial domain enhancement. The enhancement technique can sharpen the edges of the aircraft picture, highlight the outline of the aircraft, and reduce the background color to improve the contrast between the aircraft and its environment. Although picture enhancement does not increase the inherent information of the data, it increases the dynamic adjustment range of the selected characteristics and facilitates object classification.

### 2.2. BA-CNN Network

BA-CNN consists of two ResNet-34 networks as the characteristic extraction function and adds the channel attention module and spatial attention module to the two-way characteristic function, and the convolutional characteristic extracted by the two networks are bilinearly combined to achieve end-to-end weakly supervised classification. Using ResNet-34 with added hybrid attention as the characteristic extraction function, the characteristic representation capability is somewhat enhanced to pay sufficient attention to the influence of discriminative parts of objects on classification. The BA-CNN network combines the two output characteristics by outer product to generate high-dimensional bilinear characteristics.

#### 2.2.1. Network Structure

**Figure 4:**
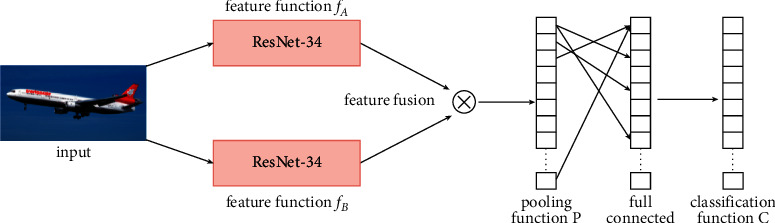
Hybrid attention network model architecture.

The hybrid attention network model uses two parallel CNNs to achieve the characteristic extraction process. In this article, the characteristic extraction process selects the ResNet34 network as the characteristic extraction function and replaces the final fully connected and Softmax layers of the two CNNs with a bilinear pooling layer, and the final bilinear characteristic representation vector is obtained by bilinear combination and pooling of the output results of the two eigenfunctions. BA-CNN network models utilize the second-order statistical information about the picture to model the combined interactions between local characteristics of translation invariance and achieve weakly supervised recognition with only picture category labels. Meanwhile, the BA-CNN network simplifies the gradient calculation, making its end-to-end network model easier to be trained, and the architecture of this network model is shown in Figure 4.

**Figure 5:**
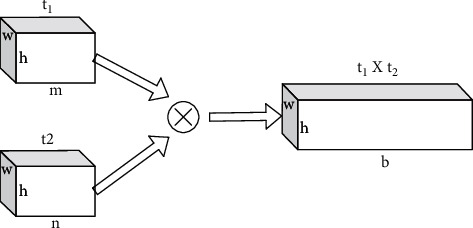
Schematic diagram of characteristic fusion.

The BA-CNN network model can be represented by a quadratic function *B=F*(*f*_*A*_,*f*_*B*_*,P,C*), where *f*_*A*_ and *f*_*B*_ are characteristic functions, *P* is the pooling function, and *C* is the recognition function. The characteristic functions *f*_*A*_ and *f*_*B*_ represent a mapping relationship *f* : *I* × *L*⟶*R*^*K*×*T*^, where *I* represents the input picture, *L* ∈ *R*^*K*^ represents the location range of the input picture, and *f* maps them into a *K* × *T* dimensional characteristic map, where *K* represents the spatial resolution size of the characteristic map and *T* denotes the characteristic channel dimension. The characteristic vectors *m* and *n* are bilinearly combined through the outer product operation (here refers to the tensor product in linear algebra [10]), and the bilinear characteristic *b*(*l*, *I*, *f*_*A*_, *f*_*B*_)=*f*_*A*_(*l*, *I*) ⊗ *f*_*B*_(*l*, *I*)=*m*^*T*^*n* is obtained, where *b* ∈ *R*^*w*×*h*×*t*1×*t*2^; *l* ∈ *L*; *L* ∈ *R*^*K*^⊗ represents the outer product operation of the vector and *T* represents the product of vector m and *n*-channel dimension *t*_1_ × *t*_2_, and the schematic diagram of characteristic fusion is shown in Figure 5.

To further obtain the picture descriptors, the pooling function *P* aggregates the bilinear characteristics at each position in the picture to obtain a global representation of the picture. One pooling approach is to sum all the bilinear characteristics cumulatively, that is, *ϕ*(*I*)=∑_*t*∈*L*_*b*(*f*_*A*_, *f*_*B*_, *l*, *I*)=∑_*t*∈*L*_*m*^*T*^*n*. The pooling function *P* obtains the vector by *ϕ*(*I*) converting the bilinear characteristic *b* into a *t*_1_*t*_2_ × 1 dimensional column vector, denoted as *xx* will be subjected to the signed open-square operation $\text{sign}\left(x\right)\sqrt{x/y}$, to which a *L*2 regularization constraint *y*/‖*y*‖_2_ is applied to obtain the final representation vector *z* that will be *z*an input to the recognition function *C*to complete the classification.

#### 2.2.2. Characteristic Extraction

The aircraft feature extraction part is mainly composed of two channels ResNet-34 as a general classification network. Compared with VGGNet, although it has a certain characteristic representation ability, it has certain limitations of discriminant local characteristic extraction in fine-grained picture recognition [11]. The more network parameters of VGGNet consume a large amount of computational resources, leading to higher memory occupancy, making the network model limited in terms of speed and precision rate, which affects the practicality.

**Figure 6:**
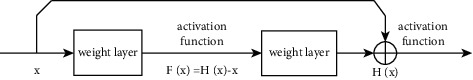
Structure of residual unit.

With the development of deep convolutional neural networks, the network depth has an important impact on the picture recognition precision rate. Usually, when there are few layers, increasing the depth can get better characteristic extraction and improve the recognition precision rate; however, when there are many layers (e.g., if more than 30 layers), continuing to increase the depth will bring higher training and testing errors, making it difficult to converge when training the network, but reducing the precision rate [12]. The main reason for the elevated error is the phenomenon of gradient disappearance and gradient explosion when increasing the number of layers, especially the problem of gradient disappearance, which prevents the gradient from being effectively updated to the shallow network of weight adjustment during back propagation. To address these problems, He et al. [13] propose a deep residual network (ResNet). Compared with other convolutional neural networks, ResNet adopts a residual learning structure to transfer the original input information directly to the next layer of the network of jump connections, while the gradients are also directly transferred to the previous layer through jump connections when back propagating. The basic structure of the residual network is the residual unit, and Figure 6 shows the structure of the residual unit.

Let *x* be the input of the residual unit and H(*x*) be the expected output of the residual unit. If *x* is passed directly to the output part as the initial result, the network only needs to learn F(x) = H(*x*) − *x* at this time, which is a basic residual unit of ResNet. This is a basic residual unit of ResNet. With this residual unit structure, ResNet is equivalent to changing the learning target for the fully output value H(x) to the distinguish between the output value and the input value H(x) −x, which simplifies the network learning target and reduces the learning difficulty. ResNet is proposed to effectively overcome the trouble of disappearing gradients in deep networks, which makes the recognition precision rate significantly improved and has good portability. BA-CNN network model using ResNet-34 has a deeper network structure and can learn local characteristics in fine-grained pictures more finely than VGGNet to improve the recognition precision rate.

Therefore, in this article, the two-way ResNet-34 was chosen as the characteristic function part of the network model, and ResNet-34 contains five groups of convolutional blocks conv1–conv5, 33 convolution layers, and one complete connected layer, total of 34 layers. The final fully connected layer is removed from the two-way ResNet-34 as the backbone of the network model, and the output characteristic dimension of the last convolutional layer of the network is 512. Compared with using the VGGNet network of characteristic extraction, the ResNet-34 networks increase the depth, while maintaining the same output characteristic dimension, avoiding the exponential increase in the characteristic dimension after the bilinear combination.

#### 2.2.3. Hybrid Attention Module

The attention mechanism is proposed by the imitation of human brain's special vision signals processing mechanism. When human is to observe and identify objects, there will be a targeted focus on target, while ignoring some significant part of the background and global information, the mechanism of selective attention in fine-grained picture recognition task rely on consistent discriminant characteristics of parts [14]. Therefore, in order to further extract judicious part characteristics, a hybrid attention mechanism is introduced in the network-using the CBAM (convolutional block attention module) algorithm to extract attention weight maps in both channel and spatial dimensions in the two characteristic functions of the backbone network, and to distribute the weights distributed in the original characteristic maps for characteristic fusion, and the fused channel attention and spatial attention modules are added between the convolutional blocks of the first network conv4 and conv5 and the second network conv2 and conv3, respectively, to obtain attention characteristics with different dimensions and more richness.

#### 2.2.4. Channel Attention Module

**Figure 7:**
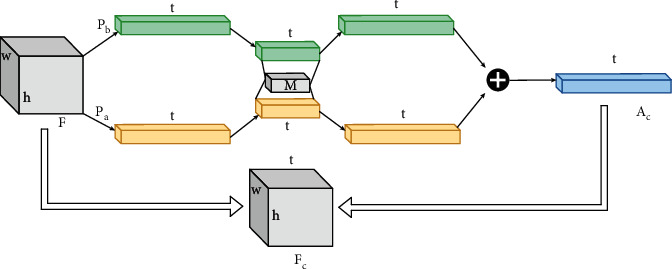
Channel attention module.

The convolutional characteristic maps produced by the characteristic functions contain different characteristic channels, and in the fine-grained picture recognition problem, each characteristic channel may represent different information in the picture, some of which contain irrelevant picture background information and are redundant. Therefore, focusing on the characteristic channel including the discriminant sites information and giving it a higher weight distribution can effectively enhance the fine-grained recognition effect. In this article, the channel attention module is added between the conv4 and conv5, and the construction of the channel attention is shown in Figure 7.

The characteristic extraction and characteristic fusion steps of the channel attention module are as follows:

1. The convolutional characteristic map *f*_*A*_ generated by the characteristic function is used as the original input F, set *F* ∈ *R*^*w*×*h*×*t*^, where *w* × *h* represents the spatial dimension of *F*, and *t* represents the number of channels. *F* is compressed in the spatial dimension, and the characteristics of the same channel are compressed into a number for extract channel attention effectively. This step can be achieved through pooling operation.
2. Take a way of pooling of multi-scale, respectively using maximum pooling functions average pooling *p*_*m*_ and *p*_*a*_ to dimension reduction of F, get two 1 × 1 × *t* characteristic vector, the size of the two input vectors in the same shared network in order to get the attention of the weight distribution channel dimension, sharing network comprises a hidden layer of multilayer perception into a unit.
3. The two output vectors after reassigning the attention weights are subjected to the corresponding element summation operation, and the combined characteristic vectors are mapped using the Sigmod activation function to generate the channel attention weights *A*_*c*_*A*_*c*_ ∈ *R*^1×1×*t*^
4. Characteristic fusion is carried out between the attention weight *A*_*c*_ and the original characteristic graph *F*. Here, a fusion method of multiplying corresponding elements is adopted to finally obtain the fused attention characteristic graph *F*_*c*_ and *F*_*c*_ ∈ *R*^*w*×*h*×*t*^. The original input characteristic F in *f*_*A*_ is replaced by *F*_*c*_ to realize the attention extraction of channel dimension.

#### 2.2.5. Spatial Attention Module

**Figure 8:**
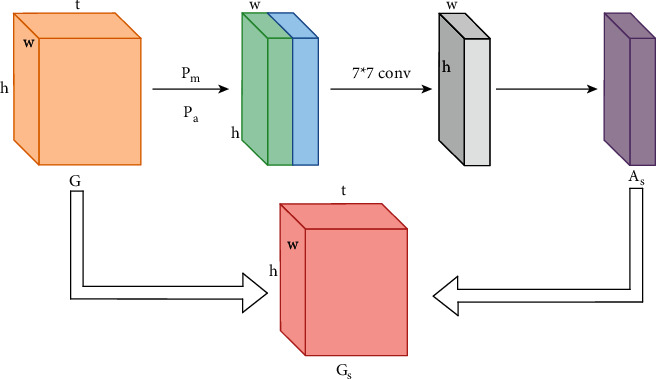
Spatial attention module.

Different from the channel attention module, the spatial attention module pays more attention to the spatial position information of the discriminant part, which is a supplement to the channel attention. Add a spatial attention module between the second channel characteristic functions conv2 and conv3, and the structure of the spatial attention is shown in Figure 8.

The steps of characteristic extraction and characteristic fusion of spatial attention are as follows:

1. The convolutional characteristic map *f*_*B*_ produced by the characteristic function is used as the original input *G* ∈ *R*^*w*×*h*×*t*^, where *w* × *h* the size of the spatial dimension represented *G* by *t* the number of channels, will be compressed *G* along the channel axis direction to extract spatial attention information, and a column of channel values is compressed into one channel, which is achieved by pooling of channel dimensions in this step.
2. The same multi-scale pooling approach is used, and the maximum pooling function *p*_*m*_ and the average pooling function are adopted *p*_*a*_*G* to decrease the dimensionality to obtain two *w* × *h* × 1 size characteristic maps, and the two characteristic maps are stitched together along the channel axis direction using the corresponding element summation way to obtain a new characteristic map of one *w* × *h* × 2 size.
3. Convolution of the spliced characteristic map using a 7 × 7 convolution kernel, again compressing its size to *w* × *h* × 1, *A*_*s*_ ∈ *R*^*w*×*h*×1^, and mapping the convolved characteristic map using the Sigmod activation function to produce a spatial attention map *A*_*s*_.
4. Finally, the spatial attention map is fused *A*_*s*_ with the original characteristic map *G* using the corresponding element dot product way to get the fused spatial attention characteristic map *G*_*s*_, *G*_*s*_ ∈ *R*^*w*×*h*×*t*^ and the original input characteristics *G* in the *G*_s_ replacement *f*_*B*_ are used to achieve attention extraction in spatial dimensions.

**Figure 9:**
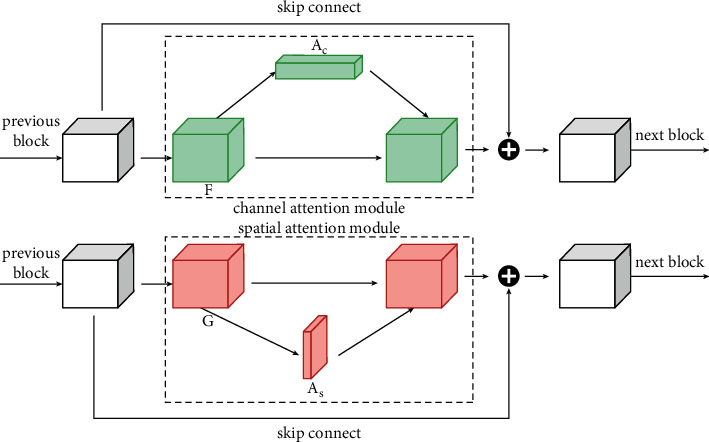
Residual attention structure of BA-CNN network model.

After adding two attention modules with two dimensions, the network acquires richer attention characteristics. The residual attention construction of the BA-CNN network model in this article is shown in Figure 9. The above two improvement methods make the network model BA-CNN with stronger local characteristic extraction ability, while fewer network parameters make the network model easier to be trained and reduce the overfitting phenomenon; at the same time, by adding attention modules of different dimensions to the residual network to obtain richer information, the BA-CNN network model can focus on and learn fine grained. This is the key to the fine performance of fine-grained recognition.

## 3. Results

### 3.1. Experimental Environment

The experimental part of BA-CNN network model is mainly divided into data preprocessing and model training. In this article, we use the PyTorch [15] as the platform, and use one NVIDIA 3090 GPU to train on the aircraft picture dataset by stochastic gradient descent method in parallel. Due to the small size of fine-grained picture dataset and limited training and testing pictures, training directly on the aircraft picture dataset may result in the network failing to converge, so the ResNet-34 network parameters pretrained on the ImageNet dataset are used for initialization, and then the network model is fine-tuned on the aircraft picture dataset. The network model was trained and optimized using the Adam [16] optimizer to train and optimize the network, with the training batch size set to 128, the first- and second-order moment estimation exponential decay rates set to 0.9 and 0.99, respectively, and the learning rate set to 0.001.

Hardware environment: Intel Core i7 12700k; 1T Memory; Nvidia RTX 3090; 32G RAM.

Software environments: CUDA Toolkit 11.1; CUDNN V11.3; Python 3.9; Pytorch 1.8.1; Windows 10.

### 3.2. Experiments and Analysis of Results

In order to comprehensively verify the effectiveness of the method in this article and to be able to better compare the recognition consequences of BA-CNN network model and its aircraft recognition algorithm, several experiments are conducted in this article as follows:

#### 3.2.1. Ablation Experiments

In this section of the ablation experimental protocol, the following network structures are compared:

1. The network-using only two-way characteristic extraction function is represented by BA-CNN (ResNet-34×2)
2. The BA-CNN (channel attention) is used to represent the network after adding only the channel attention module
3. The BA-CNN (spatial attention) is used to represent the network after adding only the spatial attention module
4. The BA-CNN (channel and spatial attention) is used to represent the network after simultaneously adding the channel attention and spatial attention to the two-way characteristic function, respectively

The experimental consequences are listed in Table 1 (backbone indicates the underlying network used for the different methods).

The results from Table 1 show that the recognition precision rate of the network after adding only the channel attention module, the spatial attention module and both modules improve by 1.0%, 1.5%, and 4.2%, respectively, over the original bilinear network model, and the highest recognition precision rate of the network is achieved after adding both attention modules simultaneously.

#### 3.2.2. Comparison Experiments

The BA-CNN aircraft recognition networks model proposed in this article does not require additional labeling information such as object labeling box and part location and only uses category labels to implement a recognition network [17] model based on weakly supervised information. The two-level attention network model, NAC[18], B-CNN, ST-CNN [19], DVAN[20], RA-CNN [21], MA-CNN, and MAMC[22]and other mainstream weakly supervised recognition algorithms in recent years, and the experimental results of the method proposed in this study and the above methods on the FGVC-aircraft dataset are compared, and the results are listed in Table 2 (backbone denotes the underlying network used by the network model and the precision rate represents the recognition precision rate).

As can be seen from the consequences of the above table, the recognition results of the method proposed in this study for the FGVC-aircraft dataset are all better than the mainstream weakly supervised methods of recent years. The consequences show that the BA-CNN networks in this article in the addition of channel attention and spatial attention modules can focus on distinguish parts in fine-grained pictures, strengthen the extraction of local characteristics, and obtain good recognition results from the fine-grained aircraft picture dataset.

## 4. Discussion

The BA-CNN hybrid attention networks model proposed in this article uses two-way ResNet-34 as the characteristic extraction function and adds a channel attention module and a spatial attention module among the residual units to achieve the introduction of a hybrid attention mechanism that strengthens the extraction of discriminative local characteristics of fine-grained pictures. The results of ablation and comparison experiments on several fine-grained picture datasets show that the way in this article can effectively improve the precision rate of the aircraft recognition model and outperforms the recognition precision rate of most of the mainstream weakly supervised algorithms in recent years. On the other hand, since the combination of bilinear characteristic vector outer product will greatly increase the characteristic dimensionality and consume computational resources, it is the progress direction of the subsequent work of this article to decrease the dimensionality of bilinear characteristics and improve the practicality of the network model, while minimizing the loss of recognition precision rate.

## Figures and Tables

**Table 1:** Experimental analysis of the ablation of the method in this article on the FGVC-Aircraft dataset.

Approach	Backbone	Accuracy (%)
BA-CNN (resnet × 2)	ResNet-34 × 2	85.0
BA-CNN (channel attention)	ResNet-34 × 2 + channel attention	86.2
BA-CNN (spatial attention)	ResNet-34 × 2 + spatial attention	86.5
BA-CNN (channel and spatial attention)	ResNet-34 × 2 + channel and spatial attention	89.2

**Table 2:** Comparison of the recognition precision rate of different weakly supervised algorithms.

Approach	Backbone	Precision rate (%)
Two-level attention	VGG19	77.9
NAC	VGG19	81.01
B-CNN	VGG-M + vgg-d	84.1
ST-CNN	Inception-v2 × 3	84.1
DVAN	VGG-19 × 3	79.0
RA-CNN	VGG-19 × 3	85.3
MA-CNN	VGG-19 × 3	86.5
MAMC	ResNet-101	86.5
BA-CNN	ResNet-34 × 2	89.2

## Data Availability

The data used to support the findings of this study are available from the corresponding author upon request.
